# *The Organon* and Materia Medica Club of the Bay Cities of California

**Published:** 1895-12

**Authors:** W. E. Ledyard

**Affiliations:** Secretary


### *THE ORGANON* AND MATERIA MEDICA CLUB OF THE BAY CITIES OF CALIFORNIA.

The regular semi-monthly meeting was held at the office of Dr. J. M. Selfridge, Oakland, July 19th, 1895.

Members present were: Drs. J. M. Selfridge, J. E. Lilienthal, W. E. Ledyard, G. J. Auger, George H. Martin, M. T. Wilson, S. E. Chapman, A. McNeil, and C. M. Selfridge. Visitor, Dr. M. F. Underwood.

The meeting was called to order at 8.15 P. M. by the President, Dr. J. M. Selfridge. The minutes of the previous meeting were read by the Secretary, Dr. W. E. Ledyard, and after two slight corrections by Dr. McNeil, were approved as read.

### Discussion upon Minutes.

Dr. Lilienthal—I would like to ask what was the reason for Dr. Selfridge giving *Tuberculinum* in that case of fibroid?

Dr. J. M. Selfridge—The first suggestion that led me to give it was the tubercular diathesis.

Dr. Lilienthal—Why I ask the question is because an article on "Nosodes" was read at the Institute at Newport. A great many prescribe nosodes because there have been symptoms of syphilis or tuberculosis, etc., before the remedy was given, but without any special evidence at the time that a nosode was indicated. The article read stated these facts. I took the stand that undoubted good results came from this method of prescribing. The nosodes ought to be proved, but if we wait until that is done we would lose many of the cases that might be cured. A great many of the drugs are proved in this way.

There is another point in the minutes that I wish to speak of, and that is in regard to "Hutchinson's teeth." They are not always a sign of syphilis, for we sometimes find them in scrofulous children when there is no syphilis at all.

Dr. McNeil—This may be so, but I have never seen it contested.

Dr. Lilienthal—Another point in regard to children who want to be carried. *Bromium* is another remedy, especially in pulmonary affections. The child wants to be carried very fast.

Dr. Ledyard—Especially in croup I have seen it mentioned.

Dr. Lilienthal—*Gelsemium*, the child is frightened; hence clings to you.

Dr. McNeil—*Cuprum*, there is a delirious fear.

Dr. Chapman—I had a very marked case of a girl four years old who cried constantly, "*Rock the bed, mamma; rock the bed, mamma!*" There was extreme photophobia and horrid irritability; the crossest child I ever saw. She had measles, combined with an acute bronchial affection.

Dr. McNeil—*Veratrum-album* would be a good remedy for such a case.

Dr. Auger—I should have given *Chamomilla*.

Dr. Chapman—*Chamomilla* hit it instantly.

Dr. J. M. Selfridge—In regard to my case from Berkeley, the woman with the fibroid, I want to state that she was in to see me to-day, and she is improving rapidly all along the line. The spots are pale instead of purple, and the pustules are drying up so that there are only a few left; the backache has gone, and the distress in urinating, frequent urging, is very materially better, and seldom troubles her except when she has been standing on her feet for some time.

Dr. Auger—I wish to ask a question a little outside of the subject under discussion. Has any one succeeded in curing tape-worm by the use of homœopathic remedies alone?

Dr. Lilienthal—I had a case which I can hardly say was a cure. A patient in New York, at the Dispensary, came to me with various symptoms. The patient did not say anything about tape-worm, nor did I suspect that tape-worm was present. I gave *Silicea*$^{30}$. In a few days the patient returned with a bottle full of tape-worm. I then looked up *Silicea*, and found that it was used for tape-worm.

Dr. Auger—I have a case in Tacoma. A woman who has been given heroic remedies. She wrote her symptoms to me, stating that portions of the worm, but not the head, had been removed. Her symptoms were great distress from gas in the stomach and abdomen, with a high fever every afternoon and evening; feet and legs cold to the knees; severe headache; rush of blood to the head; dryness of the throat. I worked the case out with Bœnninghausen's *Repertory* and Yingling's checking lists, and decided that *Sulphur* was the remedy. I sent her one dose of the 200th and blanks. I advised, also, large drinks of water. I received a letter two days ago in which she stated that nearly all of these symptoms have entirely disappeared. There is some wind still, but yet she is much better. She also stated that she had noticed pieces of the worm on her clothing. I sent blanks again, and am now waiting for the next letter.

Dr. Wilson—Dr. Pease reported a case to me which he had years ago, and which he cured with *Cina*$^{200}$.

Dr. Ledyard—Referring again to the minutes of the last meeting, I would like to ask Dr. C. M. Selfridge if the symptoms of *Mercurius* were present in the case of constipation which he mentioned?

Dr. C. M. Selfridge—No; not particularly. I gave it to see if I could cure the effects of mercurial poisoning with a high potency of the same drug.

Dr. Ledyard—That is, then, similiar to prescribing a nosode.

Dr. McNeil—I had a case of tape-worm in a girl who worked in a French laundry. These laundry girls live high. They have five meals a day, with wine. She ate a great deal; all five meals and another one at night. I told her I thought she had a tape-worm, gave her *Natrum-muriaticum*, and in a few days she brought me links of a worm.

Dr. Auger—Do you think remedies could produce tape-worm?

Dr. McNeil—It is not necessary to take a remedy long enough to find that out, as they come by spontaneous generation, though this is said to be impossible by scientists.

Dr. J. M. Selfridge—While we are talking about tape-worm you know that we have had some little controversy in the papers over here, and one homœopath replied that when he wanted to kill anything he used allopathic doses.

Dr. Chapman—This exemplifies the fact that we do not need to make a diagnosis in order to prescribe our remedy. Dr. Lilienthal cured his patient by prescribing for the symptoms present.

The President then appointed Dr. Lilienthal reader of the evening, who read from the *Organon*, Sections 223 and 224.

DISCUSSION.

Dr. Martin—This section here simply emphasizes what I stated in regard to the cause of mental symptoms in disease, moral and physical. These sentiments were promulgated over a hundred years ago, and there has not been one new point added to the care of the insane since that time. Hahnemann was the first to advocate non-restraint and kindness and gentleness in the management of these cases. It outlines the whole care in a few words, the moral, mental, and physical care. It is a whole book. It makes us realize more than anything else the greatness of the man whose work we are now studying.

Section 225 was then read.

DISCUSSION.

Dr. Auger—In the minutes just read Dr. Martin is quoted as saying that *Aurum* would always cure where there is a suicidal tendency. A case of mine in which a woman had taken one-half ounce of Carbolic Acid with suicidal intent. She is now in an insane asylum. I used *Aurum* intelligently and homœopathically without any benefit at all. I studied the case very carefully, the patient having been under my care for eleven years. She first had an attack of puerperal insanity eleven years ago, when the baby was three weeks old. She cut her own and the baby's throat. I found the bed saturated with blood. The baby died, but the mother regained her mind and was well physically for eight or ten years after. Less than a year ago she was confined again, and in about three weeks attempted to take her life. *Pulsatilla* improved at times. I

thought *Aurum* would cure her, but she is now hopelessly insane and will probably die. My experience shows that *Aurum* will not always cure where there is a suicidal tendency.

Dr. J. M. Selfridge—Some cases cannot be cured.

Dr. Lilienthal—Dr. Martin did not mean that *Aurum* will cure every case. There are other remedies with a suicidal tendency. As regards Dr. Auger's case, I think there must be some hereditary taint. Puerperal insanity is not infrequent, but it is the most readily cured of any form of insanity, and the patients usually remain well after an attack.

Dr. Auger—I think I can safely say that there is an hereditary taint in this case. Her mother died of epilepsy, and I attended her in her last illness. The father of my patient committed suicide.

Dr. Martin—In defense of the statement I made in regard to *Aurum* I will say that it will only cure where it is indicated, and the one symptom of suicidal tendency will not be enough. All of the symptoms of the patient must be taken into consideration. All cases are not curable. I wish to cite a case of puerperal mania that occurred in my practice. I confined a woman twelve years ago in Honolulu with her first child. She then had an attack of mania which lasted about three weeks. Two years later she was confined again, and had mania for about two months. Three years later was confined again, with an attack of mania lasting for about a year and a half. Two years after another child was born, with another attack of mania from which she has never recovered. Six months ago she had another child. She is now in Stockton suffering from paranoia. This shows how one form of insanity may run into another. In this case there is a deep-seated brain lesion, as there always is in paranoia.

Dr. McNeil—In suicidal tendency Dr. Talcott makes this distinction between *Aurum* and *Arsenicum:* The *Aurum* patient is sly; the *Arsenicum* patient is wild. Gallavardin, of Lyons, France, in an article on Alcoholism, says of the suicidal tendency that when they want to commit suicide by drowning, *Silicea* and *Pulsatilla* are the remedies. When they want to commit suicide by throwing themselves from a height, *Belladonna* is the remedy. There are other remedies for desire to commit suicide by poisoning. *Aurum* is only one of the remedies for suicidal tendencies.

Dr. Chapman—What form of suicide has *Aurum?*

Dr. McNeil—The patient is sly. I think they are not confined to any one method of suicide.

Dr. Lilienthal—These distinctions are very nice, but I doubt if they would work in practice. A person out of his mind does not tell you when he is going to kill himself. They will do it as opportunity offers. As a rule the people who threaten to commit suicide do not do it. Sometimes they do. Under the impulse of the moment, if the opportunity comes, they may do it. This tendency is due to a cloud which seems to hover over them.

Dr. Ledyard—As I understand, it is not that they *threaten* to do it by this way or that, but each in the way to which there is a tendency, if the opportunity offers, they avail themselves of whatever means may be at hand or what may be suggested to them—one to throw himself from a height, as Aurum ; another by drowning, Sil. or Puls., etc. *Alumina,* if he sees anything suggestive, as a knife, scissors, etc., he then has a desire for suicide.

Dr. Chapman—I differ from Dr. Lilienthal as regards patients talking about committing suicide and not doing it. I think they are very likely to talk of what is uppermost in their minds.

Dr. Lilienthal—There are other symptoms, such as delusions, hallucinations, etc., that would lead you to know that they meant what they said.

Dr. Martin—That is just the point. As Dr. Lilienthal says, the suicidal tendency is only a symptom. As Dr. Ledyard says, they will take whatever means may be at hand. A patient of my own a short time ago, when passing through the kitchen, saw the bread-knife lying on the table, took it up, and would have cut her throat if she had not been prevented. She had never manifested any suicidal tendency before.

Section 226 was then read.

DISCUSSION.

Dr. Martin—Whenever delusions are present in an insane patient, and they say they are going to kill themselves, always look out for them, for they may do it. The hysterical patient will talk about it when they have no idea of doing it. Sometimes they may do it, but it is rare. I once had an hysterical patient who often threatened to take a dose of Morphine to end her life. She took an over-dose at one time, but was rescued in time to prevent her death.

Dr. J. M. Selfridge—I knew a case of a man, a Frenchman, who was in the habit of getting drunk. Whenever that occurred his wife would thrash him, until one day he said that if she ever thrashed him again he would kill himself. She did thrash him after another of his sprees, which sobered him up, and feeling so chagrined on account of the thrashing, he took some crystals of Strychnine mixed with some whisky, drank it, and was dead in twenty minutes.

Sections 227 and 228 were next read.

DISCUSSION.

Dr. Lilienthal—I suppose there are occasions when a certain amount of force is necessary in order to get the patient to take medicine. An insane patient often has a certain fear, as of being poisoned, etc., and may refuse all food and drink. By getting control of them they may be made to take the medicine. You must get them to bed, and sometimes are obliged to fasten them down, but it can be done gently.

Dr. Martin—That can be done, and not go beyond the instructions laid down in these paragraphs, which mean that you must not use too much force; not by straps, bars, shackles, etc. Hahnemann is here railing against the brutality practiced in the English and German asylums of that day. A case of a young woman under my care, suffering from melancholia with suicidal and homicidal tendencies, three years ago. She would not take medicine, food, or water, except when she got it herself, as she was afraid of being poisoned. Therefore, I could not give her medicine without using force, and that I did not wish to do, as it would aggravate her condition and throw her into a worse state. I concluded that it was best to manage the case by keeping her quiet, and allowing her to get as much rest as possible. She lived in a flat with her parents, and a close watch was kept upon her without her being aware of it. She was not allowed to go out of the house, and in this way she obtained a great deal of rest, and in four weeks' time was much better without any medicine whatever. As soon as she would take medicine Hyoscyamus was given her, as it was the indicated remedy, and she continued to improve until in about two months she was perfectly well. There are two points illustrated by this case: first, that you have to manage these patients, and, second, that they are often benefited greatly by quiet and rest, and are more easily managed after.

Dr. Lilienthal—This recalls a conversation which I had with Dr. Talcott. He believes in rest. Patients suffering from an acute attack of insanity he puts to bed at once. He uses a restraining sheet, which is enough to keep them in bed without injuring them.

Sections 229–231 were then read.

### Discussion.

Dr. McNeil—This condition of alternating diseases is of great importance, and is illustrated by a case which I cited a few weeks ago, of alternating colic and ulcer of the leg. When we take the totality of the symptoms we have to take both of these conditions into consideration. The totality will comprehend all of the aches and pains.

Dr. J. M. Selfridge—There is one point here. Hahnemann speaks of the alternation of anti-psoric and anti-syphilitic remedies. He does not mean by that what is usually meant by the alternation of remedies. He means that you give one remedy for a certain length of time, and then later another remedy, if the symptoms have not all disappeared, for another period of time, returning again to the first remedy, if it is necessary.

Dr. Lilienthal—I may be mistaken ; but I understand that

Hahnemann meant that he had to "zigzag" his way along, giving one remedy for a certain set of symptoms for awhile, and then another remedy for another set of symptoms.

Dr. McNeil—He does not mean what is usually meant by alternation. He has been severely criticised for alternating *Aconite* and *Coffea*, but he states the *Aconite* condition when *Aconite* is to be given, and the *Coffea* condition when *Coffea* is to be given.

Sections 233 and 234 were next read.

DISCUSSION.

Dr. Ledyard—Are we to infer that he gives a solution of *Cinchona* in all intermittents?

Dr. McNeil—He uses it merely as an intercurrent remedy. The other remedies meet all the symptoms except the intermittent. Guernsey also speaks of this.

Section 235 was then read.

DISCUSSION.

Dr. McNeil—I begin to feel that I am in my native heath. I have had a greal deal of experience in intermittents. I spent a good many years in the valley of the Ohio. I have lived in these diseases. Any young man can make a reputation in handling intermittents that he could not in any other way. Quinine is unnecessary. The homœopathic remedies will be enough. The epidemic character of intermittents is spoken of. You will find one or two epidemic remedies. If there are more than one they are complimentary remedies. I have cured hundreds of cases without seeing my patient. Some could not even give an account of themselves. I did nothing but followed Hahnemann's advice. Arsenic cured one after another. In a recent case, if the remedy is given soon after an attack, there will not be another. If the case is of long standing, and Quinine has been given, it will take longer to cure. A homœopathic dose will do the work a great deal better than Quinine. We must watch the epidemic character of the disease. For a year I used *Natrum-muriaticum* with perfect success. I had one patient who would not get well. There were fever-blisters, aching pains, thirst, etc. I studied the case up in Bœnninghausen's *Intermittent*, and found that *Natrum-muriaticum* was not the only remedy for fever-blisters. *Rhus-tox.* was also mentioned. There is a striking resemblance between these two remedies. Both have fever-blisters, aching pains, thirst, etc., but *Natrum-mur.* has not the relief from motion that *Rhus-tox.* has.

Dr. J. M. Selfridge—I do not entirely agree with Dr. McNeil. *Quinine* is often the indicated remedy as well as *Rhus-tox.* or *Natrum-mur.* I had a case of a boss plumber who worked in a San Francisco basement where there were foul emanations. His was a perfect picture of marsh ague: chill, fever, perspiration in alternation, every other day. One prescription of *Quinine* $^{200}$ cured him. I did not repeat the dose, but gave blanks.

Dr. McNeil—I did not think that any one would misunderstand me. What I object to is the 6, 15, and 20-gr. doses of Quinine.

Upon motion of Dr. McNeil, seconded by Dr. Martin, the meeting then adjourned to meet the first Friday in August at the office of Dr. George H. Martin, 921 Polk Street, San Francisco, when the reading of *The Organon* would be commenced on Section 236.

Reported by Eleanor F. Martin, M. D.

W. E. Ledyard, *Secretary.*

